# Atypical Presentation of Diffuse Large B-Cell Lymphoma of the Caecum in a Young Patient: A Case Report

**DOI:** 10.7759/cureus.86150

**Published:** 2025-06-16

**Authors:** Amalan Thuraisingam, Kumail Jaffry, Brigitte Papa

**Affiliations:** 1 Surgery, Monash Health, Clayton, AUS; 2 Surgery, Monash Health, Dandenong, AUS; 3 Pathology, Monash Health, Clayton, AUS

**Keywords:** chemoradiotherapy (chemo-rt), colon mass, extranodal lymphoma of the colon, large b-cell lymphoma, primary b-cell lymphoma

## Abstract

Primary diffuse large B-cell lymphoma (DLBCL) of the colon, particularly involving the caecum, is rare and often poses a diagnostic challenge due to its non-specific presentation and the low incidence of colorectal involvement. Early recognition is crucial, as timely diagnosis and multidisciplinary management can significantly improve patient outcomes. A 29-year-old female presented with a two-week history of intermittent lower abdominal pain, progressive abdominal bloating, weight loss, night sweats, and bilateral ankle swelling over the last four weeks. Initial ultrasonography revealed a large soft tissue mass within the abdomen, initially reported as an ovarian mass; however, further evaluation, including CT imaging and colonoscopy, revealed an ulcerated, partially obstructing caecal mass. Histopathological examination of endoscopic biopsies confirmed DLBCL, non-germinal-centre B-cell subtype. PET imaging revealed multiple large, intensely fluorodeoxyglucose (FDG)-avid lymph nodes below the diaphragm forming a bulky nodal mass, along with a similarly FDG-avid caecal mass, strongly suggesting extra-nodal lymphoma involvement. The patient was subsequently managed with R-CHOP (rituximab, cyclophosphamide, doxorubicin hydrochloride (hydroxydaunorubicin), vincristine sulfate (Oncovin), and prednisone) chemotherapy under the guidance of a multidisciplinary team. Primary colonic DLBCL is rare, accounting for a small fraction of colorectal malignancies. Its non-specific symptoms, including abdominal pain, altered bowel habits, and constitutional complaints, can mimic more common gastrointestinal conditions. This case highlights the importance of maintaining a broad differential diagnosis, especially in younger patients. While imaging modalities, endoscopic assessment, and histopathology are critical for accurate diagnosis, prompt oncologic consultation and chemotherapy remain central to management. A multidisciplinary treatment approach is vital in improving patient prognosis.

## Introduction

Primary lymphoma of the colon is a rare malignant disease, though the gastrointestinal tract remains the most common site for extranodal non-Hodgkin's lymphoma (NHL) [1,2]. B-cell lymphoma specifically is the third most frequent colorectal malignancy, following colorectal carcinoma and neuroendocrine tumours, with an incidence below 0.5% [2,3]. While elderly patients account for the majority of cases, the aetiology remains unclear, and there are no well-established, standardised treatment guidelines [1,2].

Patients often present with non-specific gastrointestinal symptoms such as abdominal pain, weight loss, or bowel habit changes, leading to delayed diagnosis. Early detection is therefore critical, as management typically involves a combination of chemotherapy, radiotherapy, and sometimes surgery, all of which benefit from multidisciplinary collaboration. Within this spectrum, diffuse large B-cell lymphoma (DLBCL) is the most common NHL subtype; although it primarily arises in lymph nodes, it can manifest in the colon or rectum, accounting for 0.2-1% of colonic malignancies [1]. Given its aggressive nature, prompt recognition and appropriate patient-centred treatment are imperative to improving outcomes.

## Case presentation

A 29-year-old female patient presented to her local medical officer with a two-week history of intermittent abdominal pain, associated abdominal bloating, and pain radiating to her back that worsened with eating. She reported a 4 kg weight loss over the past four months, night sweats for the last two weeks, and bilateral ankle swelling for the last four weeks. Her past medical history was unremarkable.

On examination, vital signs were within normal limits except for a systolic blood pressure of 85 mmHg. Her abdomen was soft with generalised tenderness on palpation but no peritonism. A large palpable intra-abdominal mass was felt, and bilateral pitting oedema was present over the ankles. Initial blood tests revealed severe anaemia (Table 1), prompting the transfusion of two units of packed red blood cells (PRBCs) during initial resuscitation.

**Table 1:** Laboratory findings Reference ranges may vary depending on the laboratory. L: low; H: high; GFR: glomerular filtration rate

Tests	Patient Values	Reference Ranges & Units (Female)
Haemoglobin	63 (L)	120 - 160 g/L
White Cell Count	7.2	4.0 - 11.0 × 10^9/L
Neutrophils	6.40	2.0 - 8 × 10^9/L
Platelets	114 (L)	150 - 450 × 10^9/L
Mean Corpuscular Volume	74 (L)	78 - 98 fL
Haematocrit	0.28 (L)	0.35 - 0.48 L/L
Potassium	4.1	3.5 - 5.2 mmol/L
Urea	2.3 (L)	2.8 - 7.2 mmol/L
Creatinine	48	45 - 90 µmol/L
Estimated GFR	>90	>90 mL/min
Alkaline Phosphatase	113 (H)	30 - 110 U/L
Gamma-Glutamyl Transferase	31	5 - 35 U/L
Alanine Aminotransferase	38 (H)	5 - 35 U/L
Total Bilirubin	10	0 - 20 µmol/L
Albumin	17 (L)	35 - 50 g/L
Lactate Dehydrogenase	830 (H)	120 - 250 U/L
Haptoglobin	0.02 (L)	0.3 - 2.0 g/L

Imaging findings

**Figure 1:**
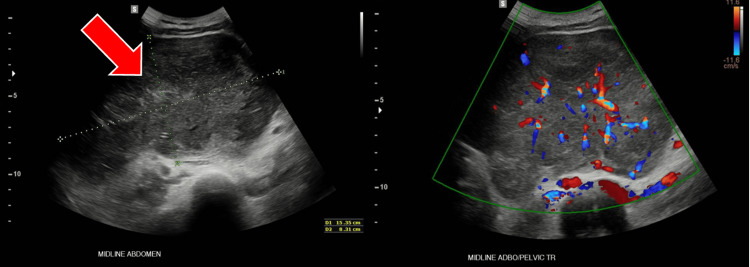
Ultrasonography of the abdomen demonstrating a large midline abdominal mass (A) Grayscale ultrasound of the midline abdomen showing a heterogeneous mass (red arrow) measuring approximately 153×83×154 mm. (B) Corresponding colour Doppler view illustrating vascularity within the lesion. The mass appears separate from the uterus and ovaries, though initially reported as an ovarian mass.

An ultrasound of the abdomen revealed a large midline abdominal mass measuring 153×83×154 mm (Figure 1).

**Figure 2:**
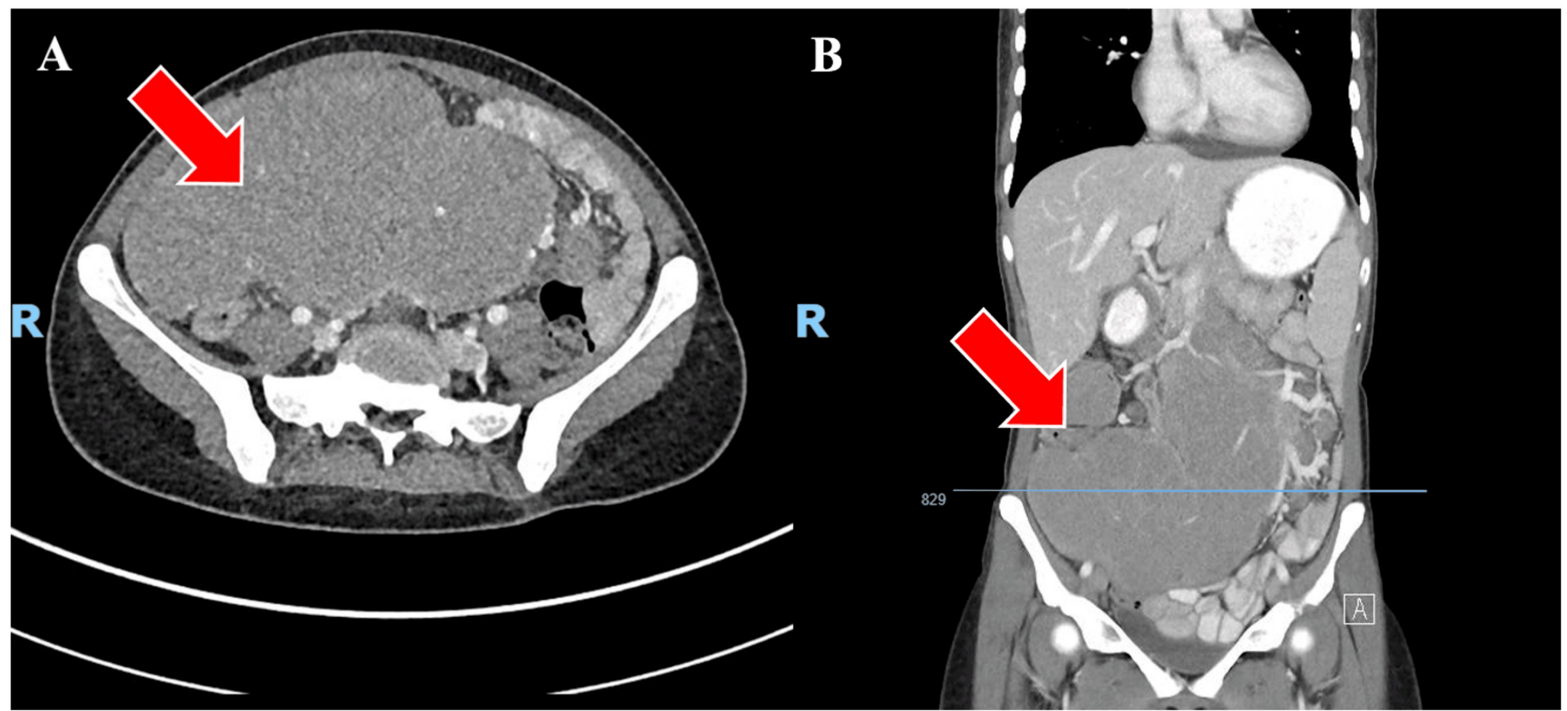
Axial (A) and coronal (B) contrast-enhanced CT scans of the abdomen and pelvis demonstrating a 19 x 18 x 9 cm mass (red arrow). It encases branches of the superior mesenteric artery and there is abnormal configuration of the ileo-caecal valve in the right side of the abdomen.

An urgent CT scan of the abdomen and pelvis revealed a large lobulated mass in the abdomen, appearing to arise from an abnormally placed ileocaecal valve (Figure 2). The differential diagnosis included a large mucocele of the appendix or pseudomyxoma.

Endoscopic and pathological findings

**Figure 3:**
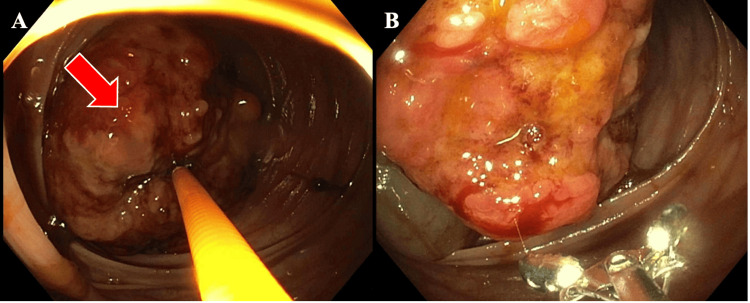
(A) Ulcerated, partially obstructing large mass (red arrow) in the caecum, without active bleeding. (B) A close-up of the malignant-appearing tumour in the colon, highlighting its extensive ulceration and irregular surface

The patient underwent an urgent colonoscopy, which revealed an ulcerated, partially obstructing large mass in the caecum (Figure 3).

**Figure 4:**
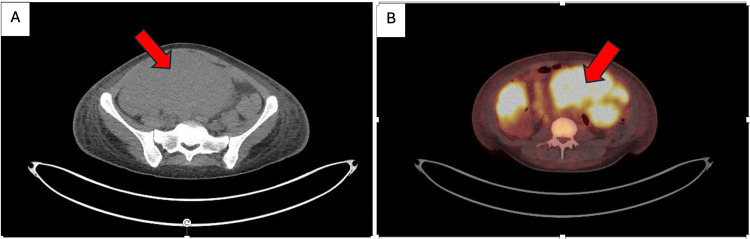
PET/CT Imaging demonstrating the hypermetabolic abdominal mass (A) Axial CT highlighting the lesion. (B) Fused axial PET/CT view confirming the hypermetabolic nature of the lesion

Her PET scan was suggestive of lymphoma with multiple, large, intensely fluorodeoxyglucose (FDG)-avid lymph nodes below the diaphragm, forming a bulky conglomerate nodal mass (Figure 4).

**Figure 5:**
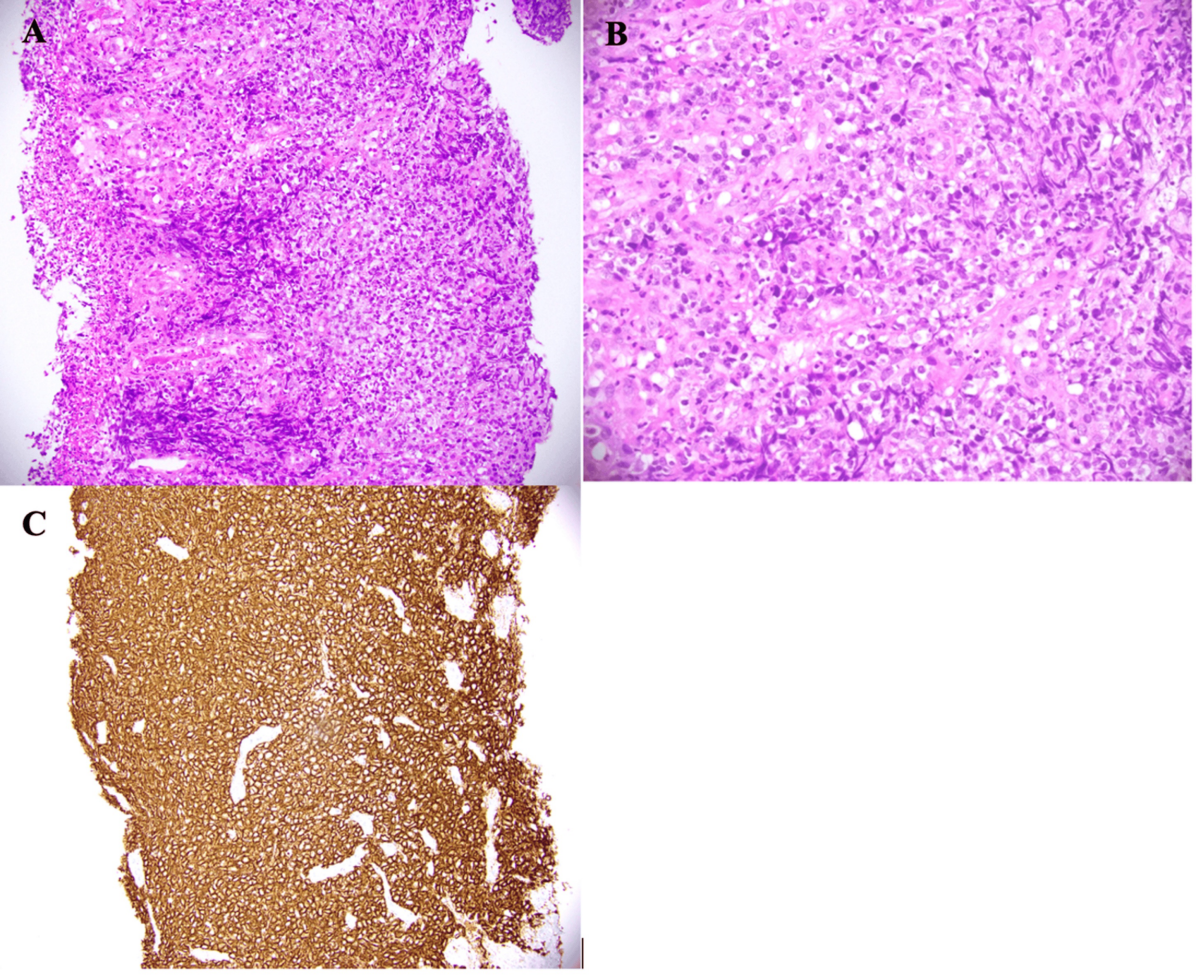
Histopathology of right colonic mass biopsy showing a diffuse infiltrate of atypical lymphoid cells with enlarged vesicular nuclei (A) 200x magnification H&E. (B) 400x magnification H&E. (C) CD20 immunostain showing diffuse strong membranous expression by tumour cells (200x magnification).

The large caecal mass was of similar intense FDG-avidity and was favoured to represent a site of extra-nodal disease. Biopsy of the right colonic mass at the time of colonoscopy revealed a diffuse infiltrate of enlarged atypical lymphoid cells with ovoid nuclei, vesicular nuclear chromatin, several conspicuous nucleoli, and clear cytoplasm (Figure 5A, 5B). Immunostains of the atypical cells showed positive expression of CD20 (Figure 5C), BCL2, BCL6, and MUM-1. The cells were negative for CD5, CD10, and cyclin-D1. Epstein-Barr virus (EBV) in situ hybridisation was negative. cMYC was positive, and Ki67 index was around 50-60%. The features were in keeping with DLBCL, non-germinal-centre B cell subtype, double expressor.

Treatment course and outcome

The patient was commenced on six urgent rounds of R-CHOP (rituximab, cyclophosphamide, doxorubicin hydrochloride (hydroxydaunorubicin), vincristine sulfate (Oncovin), and prednisone) treatment. During the first cycle of R-CHOP, she was treated for febrile neutropenia, likely due to a viral respiratory illness. A PET scan conducted after the third cycle showed a Deauville score of 3, indicating a partial metabolic response to therapy. This included the resolution of nodal disease and a significant reduction in mesenteric mass measurements. After completing the sixth cycle, the patient remained well and achieved a complete metabolic response.

## Discussion

We review a case of primary DLBCLs, a rare colonic origin cancer and the most common subtype of extra-nodal NHL, which are more aggressive than other B-cell lymphomas [2]. These tumours have been predominantly observed in immunocompromised patients such as those suffering from inflammatory bowel disease/ulcerative colitis or immunosuppression due to organ transplant or human immunodeficiency virus (HIV). It is important to note that our patient is a young female with no history of any comorbidities. The caecum has the most abundant lymphoid tissue, followed by the rectum, ascending colon, and descending colon. Therefore, the caecum is the most common extra-nodal site of involvement for colorectal lymphomas [3,4].

Primary colonic lymphomas have a male predominance, with generic symptoms including abdominal pain, weight loss, and altered bowel habits [5]. Gastrointestinal bleeding occurs in about 13-82% of patients, and the tumour reaches over 5 cm in diameter in more than 50% of patients [6]. Our patient’s CT demonstrated a large septated or loculated mass lesion in the central/upper abdomen measuring approximately 19 x 18 x 9 cm in maximum dimensions as part of her initial investigation in an emergency department. The nonspecific late symptoms due to mass effect often delay the diagnosis and worsen the overall patient outcomes. Due to the characteristic feature of perforation without desmoplastic response in lymphomas, intestinal obstruction rarely develops, and therefore, early detection of this cancer becomes difficult [7].

CT imaging modality has been the choice of initial imaging in patients with undifferentiated abdominal pain. As in this case, the patient's initial staging CT demonstrated a large septated mass lesion in the central abdomen with the abnormal configuration of the ileocaecal valve on the right side of the abdomen. It encased branches of the superior mesenteric artery and provided valuable information that the uterus in the pelvis separated from the mass, and no other axillary, hilar, or mediastinal lymphadenopathy. These CT findings were very useful in narrowing down the diagnosis to the origin of the mass from the colon. As seen in our case, colonic lymphomas are usually seen near the ileocaecal valve. If CT findings suggestive of a diffuse infiltration or bulky mass are present with the preservation of fat planes and without obstruction, lymphadenopathy, or multiple site involvement, lymphoma should be considered as the primary differential diagnosis.

Subsequent PET scan confirmed the intense FDG-avidity lesion (standardized uptake value (SUV)_max_ 13.5) from the caecum measuring up to 7 cm in length. The PET scan features of the long segment, intensely hypermetabolic, non-obstructing, circumferential wall thickening of the ascending colon and a hypermetabolic mesenteric lymph node, without any lytic/sclerotic osseous lesions, almost confirmed the diagnosis of lymphoma. It is important to note that multiple, large, intensely FDG-avid lymph nodes below the diaphragm were similar to the intense FDG-avidity of the caecal mass, confirming a site of extra-nodal disease, as we saw in our patient. These findings prompted an urgent haematology referral while waiting for the biopsy results from the colonoscopy.

No surgical resection was needed as there was no intestinal obstruction, perforation, or haemorrhage. The patient was commenced on R-CHOP, with the addition of rituximab to the CHOP regimen. Treatment with R-CHOP has remained the standard of care and is a safe, effective therapy for patients with a DLBCL [1].

## Conclusions

In the rare primary colorectal DLBCL, the majority of tumours are found within the right colon. It can be challenging when patients present with similar symptoms among some diseases, which may delay colorectal DLBCL diagnosis. Therefore, clinical suspicion and timely diagnosis are essential to provide prompt treatment and involve the multidisciplinary team in achieving long-term survival.
